# Therapy of tacrolimus combined with corticosteroids in idiopathic membranous nephropathy

**DOI:** 10.1590/1414-431X20175976

**Published:** 2017-03-23

**Authors:** W. Cui, X. Lu, X. Min, M. Liu, S. Guan, Y. Wang, M. Luo, W. Li, Q. Li, W. Dong, L. Miao, P. Luo

**Affiliations:** 1 Department of Nephrology, the Second Hospital of Jilin University, Jilin, China; 2 Department of Nephrology, Central Hospital of Jilin City, Jilin, China; 3 Department of Nephrology, General Hospital of Daqing Oil Field, Daqing, China

**Keywords:** Cyclophosphamide, Efficacy, Idiopathic membranous nephropathy, Retrospective clinical study, Tacrolimus

## Abstract

We evaluated the efficacy and safety of tacrolimus (TAC) combined with corticosteroids in treating patients with idiopathic membranous nephropathy (IMN). One hundred seventy-seven biopsy-proven IMN patients were recruited in this retrospective clinical study. Sixty patients received TAC (target blood concentration of 4–8 ng/mL) and 117 patients received daily cyclophosphamide (CYC, 100 mg) combined with prednisone. Remission rates at the end of the first, second and third month in the TAC group were significantly higher than that in the CYC group (1st: 35.0 *vs* 19.7%, P<0.05; 2nd: 56.7 *vs* 38.5%, P<0.05; 3rd: 76.7 *vs* 59.0%, P<0.05). In the first 3 months, daily urinary protein and serum albumin in the TAC group obtained a better improvement than that in the CYC group (P<0.05). At the end of the sixth and the twelfth month, the remission rates, daily urinary protein and serum albumin were all comparable between the two groups (P>0.05). No significant difference of relapse rate between the groups was found (16.3 *vs* 12.0%, P>0.05). Patients were more likely to develop glucose intolerance in the TAC group. The TAC regimen obtained more benefits in treating IMN patients, especially in the first 3 months, than the CYC regimen.

## Introduction

Idiopathic membranous nephropathy (IMN), one of the most common causes of nephrotic syndrome, is characterized by capillary wall thickening, normal cellularity, IgG and C3 along capillary walls on immunofluorescence, and subepithelial deposits on electron microscopy. Although one third of the patients with IMN achieve a spontaneous remission, the renal function continues to deteriorate in others. In 2009, Beck et al. first reported that the main pathogenic antibody of IMN targets m-type phospholipase A2 receptor (1). This point of view was further confirmed by a recent meta-analysis (2). These studies provided nephrologists with a theoretical basis for the treatment of IMN with immunosuppressive therapy. According to Kidney Disease Improving Global Outcomes (KDIGO) guidelines, a 6-month course of alternating monthly cycles of oral and intravenous corticosteroids, and oral cyclophosphamide (CYC) was recommend as an initial therapy for IMN (3). However, since numerous adverse events due to the long-term use of CYC, such as irregular menstruation, cancer risk, gonadal and bladder toxicity, and infections (4,5) have been reported, alternative regimens for the initial therapy of IMN are desired.

As one of the calcineurin inhibitors, tacrolimus (TAC) has been used in primary glomerulopathy for many years because it can inhibit the protein phosphatase activity of calcineurin, leading to suppression of the nuclear translocation of various genes and to inhibition of T cell activation (6). Our previous study showed that a low dose of TAC in combination of corticosteroids was effective for mild mesangial proliferative glomerulonephritis (7). Additionally, the efficacy of TAC in the treatment of focal segmental glomerulosclerosis was also reported (8). The therapeutic effect of TAC on membranous glomerulonephritis was initially investigated in rats (9); then the first randomized controlled trial, where TAC monotherapy was studied in IMN patients, was carried out in 2007 (10). Praga et al. (10) found a significantly larger number of remissions in TAC-treated patients compared with placebo patients during the 18-month follow-up, suggesting that TAC was a very useful therapy for IMN patients. One year later, a prospective non-randomized cohort study comparing two regimens, TAC plus prednisone and CYC plus prednisone, was performed for treating IMN (11). Li et al. (11) concluded that both regimens were effective for IMN patients in achieving remission of severe proteinuria, and a faster remission was found in the therapy combining TAC and prednisone. Then, Chen et al. (12) demonstrated the effectiveness of TAC for treating IMN once again in a multicenter randomized controlled trial organized in China. Based on these studies, TAC combined with corticosteroids was recommend by KDIGO guidelines as an alternative regimen for the initial therapy of IMN (3).

Up to now, most studies focusing on the effects of TAC on IMN had a small sample size (11–16). Thus, in order to increase the statistical efficiency, in the present study, we retrospectively analyzed data from 177 IMN patients with massive proteinuria treated with TAC or with CYC.

## Material and Methods

### Patients

This was a retrospective clinical study conducted at the Second Hospital of Jilin University. IMN patients hospitalized during January 2009 to June 2015 were recruited. The inclusion criteria were as follows: 1) a biopsy-proven IMN (stage I–IV); 2) massive proteinuria (urinary protein >3.5 g/day) accompanied by hypoalbuminemia (serum albumin <30 g/L); 3) serum creatinine lower than 176 μmol/L; and 4) age between 16 and 85 years. The following exclusion criteria were applied: 1) coexistence of other severe renal diseases; 2) life-threatening complications such as severe infections or heart failure; 3) HBV- or HIV-positive serology; 4) malignancy, or other contraindications of corticosteroids and immunosuppressive agents; 5) diabetes mellitus; 6) pregnancy or lactating; 7) hypersensitivity to macrolides medication; 8) received corticosteroid and/or immunosuppressive agents before our study.

### Renal tubulointerstitial lesion score

Tubulointerstitial lesions included interstitial inflammatory cell infiltration, interstitial fibrosis and tubular atrophy. Renal tubulointerstitial lesion score evaluation standard was no lesion, 0 point; lesion area <25%, 1 point; lesion area ranging from 25 to 50%, 2 points; lesion area >50%, 3 points. Total score ranged from 0 to 9 points.

### Medication regimens

Time elapsed between diagnostic renal biopsy and patients' inclusion in our protocol was specified as time elapsed from kidney biopsy to present treatment. One hundred seventy-seven patients, who were assigned either to the TAC (n=60) or CYC group (n=117), were followed-up for 12 months.

Patients in the TAC group received oral tacrolimus, starting with a dose of 0.05 mg·kg^-1^·day^-1^, divided into two doses at 12-h intervals. We adjusted the dosage according to the whole blood concentration, with a target of 4–8 ng/mL throughout the first 6-month therapy period, and kept the maximum dose to no more than 0.15 mg·kg^-1^·day^-1^. During the next 6 months, the dosage of TAC was reduced at intervals of 4–8 weeks to a level of about 50% of the starting dose. Since this dosage was very low, blood concentration of TAC was no longer monitored. All the patients received oral prednisone 0.5 mg·kg^-1^·day^-1^ (no less than 30 mg/day) for 8 weeks, and were gradually tapered off (5 mg reduction for every 4 weeks) until 10 mg/day; this dosage was maintained throughout the remainder of the 12-month therapy period.

In the CYC group, patients were given a daily oral CYC dose of 100 mg for 3 months (accumulated dosage was 9 g). All the patients received an oral prednisone dose of 1.0 mg·kg^-1^·day^-1^ (no more than 60 mg/day) for 8 weeks, and were gradually tapered off (5 mg reduction for every 4 weeks) until 10 mg/day, a dosage that was maintained throughout the remainder of the 12-month therapy period. The dose of renal angiotensin system (RAS) inhibitors remained unchanged during this study. Target blood pressure was 130/80 mmHg.

### Follow-up

Follow-up visits or telephone follow-ups were performed at 1, 2, 3, 6, and 12 months after the initiation of the immunosuppressive therapy described above. At each follow-up visit, standard complete blood count, serum creatinine, glucose, aminotransferase, total proteins, albumin, cholesterol, triglycerides, urinary analysis, 24-h urine protein, as well as side effects were recorded. Trough levels of TAC were examined for the first 6 months.

### End points

The primary end point was complete remission (CR) or partial remission (PR). CR was defined as proteinuria <0.3 g/day plus normal creatinine concentration, while PR was defined as proteinuria 0.3–3.5 g/day and 50% lower than baseline proteinuria, plus stable renal function. Patients who did not meet the above CR or PR criteria were regarded as non-responders (NR). Relapse was defined as an achievement of CR or PR, followed by a new nephrotic syndrome, without remission two weeks after removing the triggering factors (such as infection). The secondary end point was proteinuria, serum albumin and renal survival, which was defined as a serum creatinine concentration 50% higher than the baseline value. Time to remission was defined as the mean time to reach remission (PR or CR).

### Statistical analysis

Differences of quantitative parameters between groups were assessed using the *t*-test as appropriate. Qualitative results were compared using the chi-square test. P<0.05 was considered to be statistically significant. Analyses were performed with Prism statistical software package (version 5.0, USA).

## Results

### Demographic data

One hundred seventy-seven patients with biopsy-proven IMN were included. Baseline characteristics of the patients were compared, and no significant differences were found between the TAC group and the CYC group (Table 1).

**Table 1:**
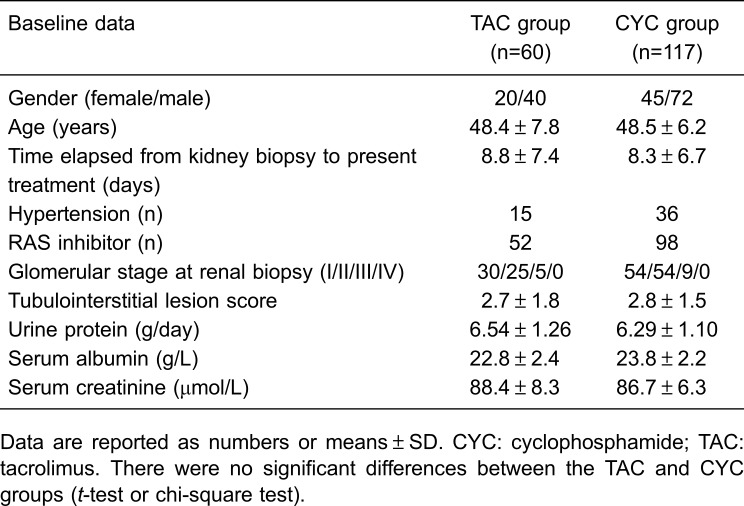
Demographic, histological and laboratory characteristics of patients at baseline.

### Blood concentration of TAC

During the first 6 months, the trough level of TAC was 5.62±0.59 ng/mL (range 2.41–11.38 ng/mL), and most TAC concentrations fell in the range of 4-8 ng/mL. During the second period of 6 months, blood concentration of TAC was no longer monitored.

### End points

The primary end point was CR or PR (Table 2). After 6 months of therapy, 49 (81.7%) of 60 patients from the TAC group achieved remission, and 86 (73.5%) of 117 patients from the CYC group reached the primary end point. No significant difference was found between the two groups (P=0.23). Because recurrence occurred in both groups during the last 6 months, the remission rate decreased to 70.0% in TAC group and 70.1% in CYC group at the end of the twelfth month. The numbers of patients with CR in the TAC and CYC groups were 31 and 41, respectively. The percentages of remission in the TAC and CYC groups were, respectively, 35.0 and 19.7% by 1 month (P=0.03), 56.7 and 38.5% by 2 months (P=0.02), 76.7 and 59.0% by 3 months (P=0.02). There were 11 (18.3%) and 31 (26.5%) NR patients in the TAC and CYC groups, respectively, by 6 months, where the mean daily urinary protein was 0.98±0.61 g and 1.83±0.68 g, respectively. There were 10 (16.7%) and 21 (17.9%) NR patients in the TAC and CYC groups, respectively, by 12 months, where the mean daily urinary protein was 0.84±0.41 g and 1.44±0.42 g, respectively (Table 2).

**Table 2:**
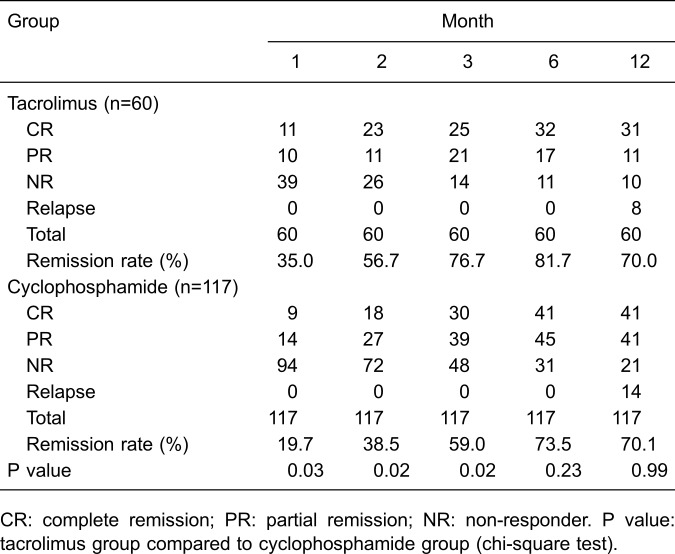
Remission rates in idiopathic membranous nephropathy patients treated with tacrolimus or cyclophosphamide.

**Figure 1:**
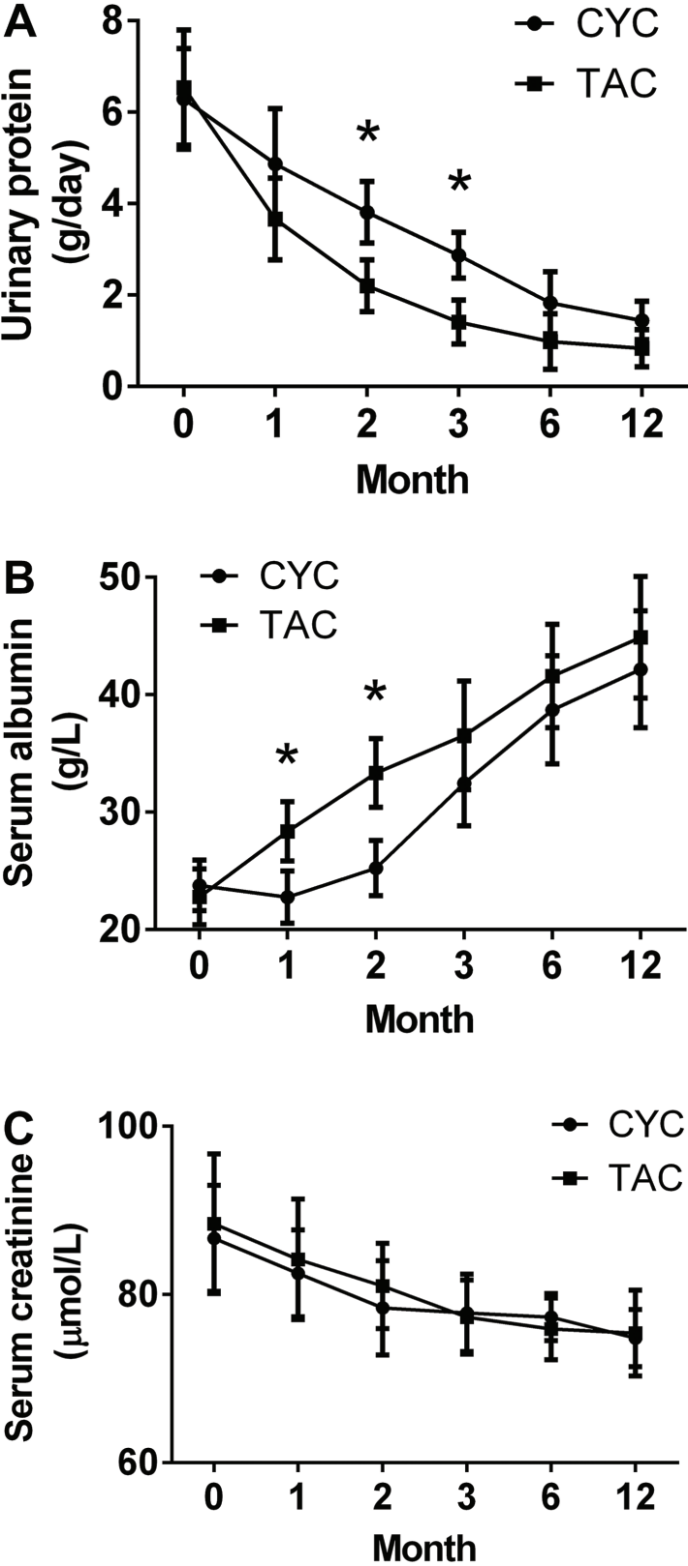
Evolution of the secondary end points in two groups. Daily urinary protein (*A*), serum albumin (*B*), serum creatinine (*C*). CYC: cyclophosphamide; TAC: tacrolimus. *P<0.05, TAC group *vs* CYC group (*t*-test).

From baseline to the end of the twelfth month, the mean daily urinary protein decreased from 6.54 to 0.84 g in the TAC group and from 6.29 to 1.44 g in the CYC group (Figure 1A). The mean serum albumin increased from 22.8 to 44.9 g/L in the TAC group and from 23.8 to 42.2 g/L in the CYC group (Figure 1B). Detailed daily urinary protein and serum albumin levels during the 12-month treatment period are shown in Figure 1A and B.

Relapse occurred in 8 patients in the TAC group and in 14 patients in the CYC group during the 12-month treatment period. No significant difference of relapse rate between the TAC (16.3%) and CYC (12.0%) groups was found (P>0.05). As an important note, most relapses took place in patients with PR; the proportion of relapses in PR patients and CR patients was 68.2 and 31.8%, respectively.

### Evaluation of renal function

Monitored during the 12-month period of treatment, serum creatinine had the tendency to decrease (Figure 1C). For TAC and CYC groups during the follow-up period, the doubling of serum creatinine with respect to baseline was observed in 2 and 7 patients, respectively. No significant difference in renal dysfunction rate between the TAC and CYC groups was found (3.3 *vs* 6.0%, P>0.05).

### Side effects

Some patients experienced non-severe adverse events during the 12-month treatment. Impaired glucose tolerance was found in 16 patients from the TAC group and 17 patients from the CYC group (26.7 *vs* 14.5%, P<0.05). Irregular menstruation was found in 1 patients from the TAC group and 12 patients from the CYC group (1.7 *vs* 10.3%, P<0.05). In addition, similar infection, mild gastrointestinal symptoms, liver enzyme increase, blood leucocyte decrease and new-onset hypertension were found in both groups (Table 3).

**Table 3:**
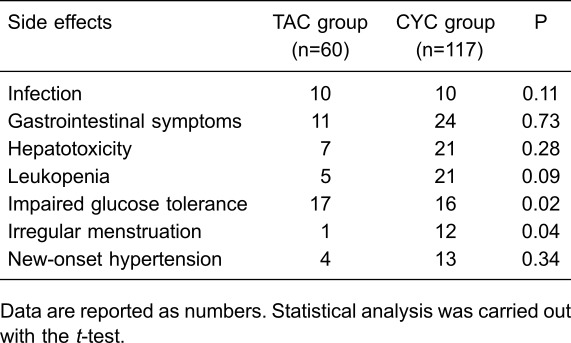
Adverse effects in idiopathic membranous nephropathy patients treated with tacrolimus (TAC) or cyclophosphamide (CYC).

## Discussion

Treatment of IMN remains a challenge for nephrologists. For those patients with high risk, corticosteroids in combination with an immunosuppressant are recommended (3). The most commonly used immunosuppressive agents for treating IMN are alkylating agents, such as chlorambucil and CYC. Considering the unsatisfactory results and side effects of alkylating agents, calcineurin inhibitors like TAC emerged as an alternative therapeutic option for patients with IMN (17). Previous studies reported that TAC, either monotherapy or in combination with corticosteroids, was effective in treating IMN patients (10–14 [Bibr B15],^18^). More importantly, several studies also suggested a valuable role of TAC in corticosteroid-resistant primary glomerulopathy (19,20). However, all the above studies had small sample sizes which decrease the statistical power.

In the current study, we recruited 177 IMN patients who were prescribed either TAC or CYC in addition to corticosteroids for a period of 12 months. Twelve months after the initiation of treatment, decreased urinary protein, increased serum albumin, stable renal function, together with improvement of edema were observed in most participants. The achievement of CR or PR in IMN patients could significantly reduce the risk of renal failure (21). Our results showed that compared to the CYC group, patients in the TAC group achieved a similar remission rate after 12 months of treatment (70.0 *vs* 70.1%). Our remission rate at 12 months was comparable to a previous study (13). Ning and Sanchez (22) reported that besides the inherent immunosuppressive effect, TAC could also enhance the immunosuppressive action of steroids by potentiating glucocorticoid receptor affinity. This may be one of the underlying mechanisms by which this combination therapy achieved such favorable results in treating IMN nephrotic syndrome. Moreover, the percentage of remission by the first 3 months in the TAC group was significantly higher than that in the CYC group. Our remission rates were comparable to those of a recent study (11). Of note, the mean daily urinary protein for patients with NR was no more than 4.0 g by 3 months; therefore, we did not change our original therapy regimen. At the end of the twelfth month, the mean daily urinary protein remained fluctuating at an acceptable range.

In the present study, time to remission (mean time to reach remission) of TAC was 2.26 months, which was similar to 2.64 months reported in another trial studying the effect of a low dose of TAC (2-4 ng/mL) on corticosteroid-resistant IMN (16). In our study, the TAC blood concentration ranged between 4–8 ng/mL, which was higher than 2–4 ng/mL reported in the trial of He et al. (16). It was disappointing that the normal TAC concentration could not improve the onset time. Thus, how to shorten the onset time of TAC in treating corticosteroid-resistant IMN patients needs to be further explored.

It is well known that the classic predictive risk factors for renal damage in IMN include male gender, old age, level of persistent proteinuria, renal tubulointerstitial lesion score, as well as impaired renal function at baseline (23,24). All these items were similar between the two groups in the present study. Of note, the use of RAS inhibitors, which is strongly recommended in KDIGO guidelines as a first-line choice to treat IMN (3), was not evaluated in most previous randomized controlled trials (11,14,16). In the present study 52 (86.7%) and 98 (83.8%) patients from the TAC and CYC groups were prescribed RAS inhibitors, respectively. The use of RAS inhibitors in both groups was also balanced. The reasons for which patients in each group did not use RAS inhibitors were: 1) their blood pressures were too low to tolerate these medicines; and 2) the patients' compliance was low. Recently, some new markers, such as angiopoietin-like-4 (25) and m-type phospholipase A2 receptor (26), were reported to predict the clinical response in patients with IMN. In a recent study evaluating the therapeutic effect of TAC on IMN, a very good association between m-type phospholipase A2 receptor antibody and proteinuria was found, and this specific antibody was recommended to be regularly monitored during the clinical follow-up (13). In the present study, m-type phospholipase A2 receptor antibody was not evaluated, which is an important limitation.

Interestingly, significant differences in serum albumin level and urinary protein level were found in the first and second months, respectively. This phenomenon suggested that patients from the TAC group showed a rapid increase of their serum albumin levels before obvious reduction of urinary protein. Our finding agreed with a previous study (16). The underlying mechanism might be the protective effect of TAC on hepatocytes and its inhibitory effect on interleukin-6; both actions decrease hepatic albumin secretion (27).

Relapse is an important issue that should be considered when using TAC (28). The relapse rate in the current study was 16.3 and 12.0% in the TAC and CYC groups, respectively. Relapse rate of TAC was not higher than CYC, which is the classical medicine used for treating IMN. The current result was similar to that reported in the study of Chen et al. (12). Moreover, in our study, we found that most relapses occurred in patients with PR. We tried to further evaluate the correlations between the urinary protein levels and the risk of relapse by comparing the baseline urine protein between relapse cases (n=22) and non-relapse cases (n=124). Compared to non-relapse cases, higher but not significant urinary protein level was found in relapse cases (6.89 *vs* 6.12 g/day, P=0.17). Different studies (29,30) also claimed that relapses occurred more frequently in IMN patients with PR and that they can be reduced by a longer TAC tapering period. The tapering period in the current study lasted 6 months. In order to further reduce recurrence, prolonging the maintenance stage with low doses of TAC was recommended. However, it remains unclear how long TAC should be used.

Another issue in using TAC is the occurrence of adverse events. The main side effects that occurred in the TAC group were impaired glucose tolerance (17/60). For patients who suffered from impaired glucose tolerance, blood glucose was monitored and controlled by oral anti-diabetic medication, if necessary. No other severe adverse events were found, suggesting that TAC combined with prednisone was a safe regimen for treating patients with IMN.

In conclusion, compared to CYC plus prednisone regimen, TAC in combination with prednisone obtained more benefits in treating IMN patients, especially in the first 3 months. In order to reduce relapses, a prolonged maintenance of a low TAC dose might be required.
